# Giant Magnetoresistance-based Biosensor for Detection of Influenza A Virus

**DOI:** 10.3389/fmicb.2016.00400

**Published:** 2016-03-29

**Authors:** Venkatramana D. Krishna, Kai Wu, Andres M. Perez, Jian-Ping Wang

**Affiliations:** ^1^Department of Veterinary Population Medicine, College of Veterinary Medicine, University of Minnesota, St. PaulMN, USA; ^2^Department of Electrical and Computer Engineering, University of Minnesota, MinneapolisMN, USA

**Keywords:** giant magnetoresistance, biosensor, magnetic nanoparticle, GMR chip, influenza A virus

## Abstract

We have developed a simple and sensitive method for the detection of influenza A virus based on giant magnetoresistance (GMR) biosensor. This assay employs monoclonal antibodies to viral nucleoprotein (NP) in combination with magnetic nanoparticles (MNPs). Presence of influenza virus allows the binding of MNPs to the GMR sensor and the binding is proportional to the concentration of virus. Binding of MNPs onto the GMR sensor causes change in the resistance of sensor, which is measured in a real time electrical readout. GMR biosensor detected as low as 1.5 × 10^2^ TCID_50_/mL virus and the signal intensity increased with increasing concentration of virus up to 1.0 × 10^5^ TCID_50_/mL. This study showed that the GMR biosensor assay is relevant for diagnostic application since the virus concentration in nasal samples of influenza virus infected swine was reported to be in the range of 10^3^ to 10^5^ TCID_50_/mL.

## Introduction

Influenza viruses belong to the family *Orthomyxoviridae*, which are enveloped single strand negative sense RNA viruses with segmented RNA genome. Based on their matrix (M) and nucleoprotein (NP), influenza viruses are classified into type A, B, or C. Influenza A viruses (IAVs) are further classified into subtypes based on their surface glycoproteins, hemagglutinin (HA), and neuraminidase (NA). IAV is a common respiratory pathogen infecting many hosts including humans, pigs (swine influenza virus or SIV) and birds (avian influenza virus or AIV). In addition to SIV, pigs are susceptible to infection with influenza viruses of human and avian origin and this is believed to contribute to novel reassortant influenza viruses with pandemic potential (Kida et al., 1994). Surveillance of swine and avian influenza viruses in the wild, in farms, and in live bird markets is critical for detection of newly emerging influenza viruses with significant impact on human and veterinary public health. Rapid, sensitive, and reliable method for detection of IAV in the environment, tissues and body fluids is important for controlling the infection and reducing the impact of possible influenza pandemic by early detection and rapid intervention. Currently, laboratory diagnosis of IAV relies on isolation of virus in embryonated chicken eggs or cell culture, detection of viral antigens, serological tests to detect virus specific antibodies, and detection of viral RNA by reverse transcription-quantitative polymerase chain reaction (RT-qPCR; Lee et al., 1993; Townsend et al., 2006; Leuwerke et al., 2008; Chen et al., 2011). Virus isolation is sensitive method and considered gold standard for virus diagnosis (Amano and Cheng, 2005), however, this labor intensive technique requires average of 3–7 days to obtain the results (Ellis and Zambon, 2002). Detection of viral antigens and serological test for antibody detection are either poor in specificity or low in sensitivity. Although, RT-qPCR is highly sensitive and specific method, its requirement for expensive laboratory instruments and technical expertise (Ellis and Zambon, 2002; Payungporn et al., 2006) in addition to longer time for the completion of the test, as it involves RNA extraction step, limits its application in the field. The objective of this study is to develop sensitive and specific method for detection of swine influenza viruses with minimum sample handling and laboratory skill requirements.

Various technologies have been developed for rapid, sensitive, and specific detection of virus using nanotechnology-based approaches (Lee et al., 2011; Nidzworski et al., 2014). These technologies use nanoparticles in combination with electrical or electrochemical detection (Patolsky et al., 2004; Tam et al., 2009; Shirale et al., 2010; Driskell et al., 2011; Li et al., 2011; Singh et al., 2014). To date, chip-based giant magnetoresistance (GMR) spin valves along with magnetic nanoparticles (MNPs) have become a powerful tool for high sensitivity, real-time electrical readout, and rapid biomolecule detection (Baselt et al., 1998; Rife et al., 2003; Graham et al., 2004; Schotter et al., 2004; Millen et al., 2005; Loureiro et al., 2009, 2011; Gaster et al., 2011; Wang et al., 2014). The fabrication and integration of GMR biosensors are compatible with the large multiplex technology and the current Very Large Scale Integration (VLSI) technology (Wang et al., 2015) so it is possible to lower down the cost if the mass production is carried out. Moreover, GMR chips can be integrated with not only electronics but also microfluidics for immunoassay applications (Xu et al., 2008; Zhi et al., 2012). In addition, GMR biosensors are matrix-insensitive (Zhang and Zhou, 2012) and therefore their performance are very robust and not affected by environmental factors such as temperature and pH.

Giant magnetoresistance-based immunoassay detection is based on the principle that stray field from MNPs that bound on sensor surface will alter the magnetization in free layer (**Supplementary Figure S1**), thus changing the resistance of GMR sensors (Baibich et al., 1988; Binasch et al., 1989). A higher number of MNPs bound to GMR sensors per unit area leads to a higher detection signal. GMR sensors have been utilized previously in biomolecule and chemical detection (Srinivasan et al., 2009, 2011; Zhi et al., 2012). Unlike fluorescent labels used in immunofluorescence methods, MNPs do not bleach (Eickenberg et al., 2013). In addition, there is no ferromagnetism property in biological samples, allowing the detection of magnetic signals with less background noise (Zhang et al., 2013). Nowadays, the size of MNPs can be controlled to the identical size as the biomolecules to which they will interact with (Hsing et al., 2007). Furthermore, labeling large molecules as well as nano- or micro-particles with small biomolecules can be successfully realized (Hsing et al., 2007; Ladj et al., 2013; Zhou et al., 2015).

In the present study, we demonstrated sensitive detection of influenza virus using GMR biosensors. The virus type specific broadly reactive monoclonal antibodies to NP employed in this study were able to detect IAV of swine and human origin in direct antigen capture enzyme linked immunosorbent assay (ELISA). Using swine influenza virus H3N2v as a representative virus we found the limit of detection of GMR biosensor assay was 1.5 × 10^2^ TCID_50_/mL virus. Comparison of GMR biosensor-based detection with antigen capture ELISA showed that GMR biosensor was more sensitive. In addition, GMR biosensor-based assay allows for a real time measurement of signals. The signals are captured and processed immediately as it is generated and can be monitored continuously without operator intervention.

## Materials and Methods

### Viruses

The human pandemic influenza A/California/04/2009 (H1N1 CA/09), the swine influenza viruses A/Sw/Iowa/73 (H1N1 IA/73), A/Sw/Illinois/2008 (H1N1 IL/08), and A (H3N2) variant virus (H3N2v) were obtained from the University of Minnesota Veterinary Diagnostic Laboratory (St Paul, MN). Viruses were propagated in Madin-Darby canine kidney (MDCK) cells (ATCC CCL-34) in Dulbecco’s modified Eagle medium (DMEM) containing 0.5 μg/mL TPCK-trypsin (Worthington Biochemical Corporation, Lakewood, NJ, USA) and purified from the clarified cell culture supernatants by ultracentrifugation through a 30% (w/v) sucrose cushion and stored in aliquots at -80°C. Culture supernatant from un-infected MDCK cells were processed similarly to use for mock virus preparation. The concentration of purified virus was determined by TCID_50_ assay. For immunoassays the virus was inactivated at 60°C for 1 h. To disrupt the virus particles, the mock and virus preparation were treated with 1% IGEPAL CA-630 (Sigma-Aldrich, Product No. I8896) for 10 min at 37°C.

### GMR Chip Fabrication and Sensor Array Structure

The multilayer GMR spin valve films with top–down structure of Ta (50 Å)/NiFe (20 Å)/CoFe(10 Å)/Cu(33 Å)/CoFe(25 Å)/IrMn(80 Å)/Ta (25 Å) were deposited by a Shamrock Magnetron Sputter System onto Si/SiO_2_ (1000 Å) substrate at the University of Minnesota. A 4-inch GMR wafer containing 21 usable chips is manufactured by photolithography, ion beam milling, and electron beam evaporation techniques. An 18 nm thick Al_2_O_3_ layer was coated onto chip surface by atomic layer deposition (ALD) followed by a 20 nm SiO_2_ layer by plasma-enhanced chemical vapor deposition (PECVD) in order to prevent current leakage and in the meanwhile SiO_2_ layer paves the way for future surface functionalization.

**FIGURE 1:**
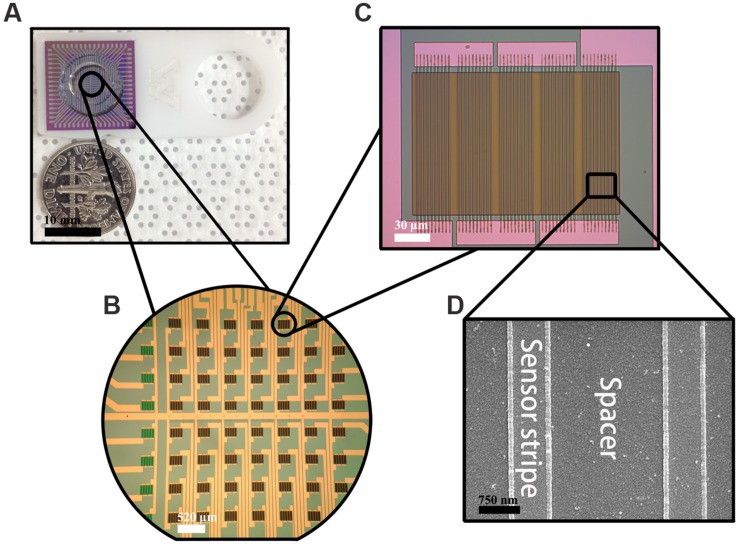
**Fabricated giant magnetoresistance (GMR) chip.** **(A)** GMR chip; **(B)** 8 × 8 sensor array; **(C)** one GMR biosensor structure; **(D)** GMR strip structure.

Each GMR chip is in the size of 16 mm × 16 mm with 8 × 8 sensor array in its center (**Figures 1A,B**). Each sensor is in the size of 120 μm × 120 μm containing five GMR strip groups connected in series and each group contains 10 GMR strips connected in parallel (**Figure 1C**). Each strip with the size of 120 μm × 750 nm is separated by 2 μm (**Figure 1D**). All the GMR chips were annealed at 200°C under an applied magnetic field of 0.5 Tesla along the minor axis (**Figure 1C**) for 1 h then naturally cooled down to room temperature in order to fully align the magnetization in the pinned layer.

### GMR Biosensor Surface Functionalization

**FIGURE 2:**
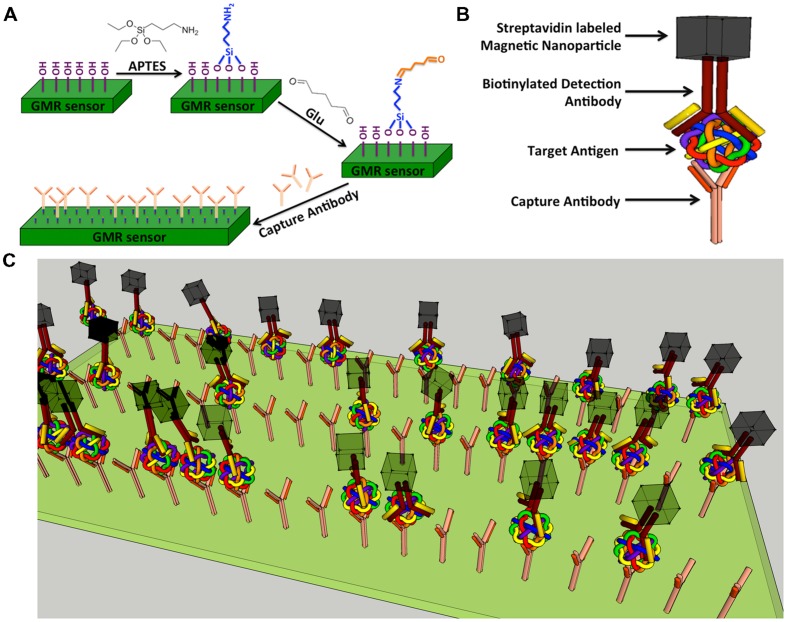
**Schematic representation of GMR biosensor.** **(A)** Schematic diagram of GMR biosensor surface functionalization. **(B)** Schematic drawing of a typical sandwich structure; **(C)** Schematic illustration of influenza A virus detection.

Giant magnetoresistance chips are first exposed to ultraviolet light and ozone (UVO) for 15 min to remove organic material from the sensor surface as well as to expose the hydroxyl group bonding sites. Each chip is then soaked in 5 mL anhydrous toluene mixed with 1% of 3-aminopropyltriethoxy silane (APTES) for 1 h at room temperature to allow APTES to covalently bind to the hydroxyl group (**Figure 2A**) from silica layer that is on-top-of GMR biosensors. Chips are thoroughly rinsed with acetone followed by ethanol and dried with nitrogen gas. The surface of APTES modified chips contain amino groups. To attach aldehyde groups onto sensor surface, the 64-sensor-array area of each chip is covered with 5% glutaraldehyde (Glu) solution (100 μL) and incubated at room temperature for 5 h under a relative humidity of ∼97%. The terminal aldehyde groups generated on the sensor surface allow subsequent covalent bonding of biomolecules containing amino groups onto GMR sensor (Wang et al., 2013, 2014, 2015).

### Influenza A Virus Immunoassay

3-Aminopropyltriethoxysilane–Glu modified GMR sensors were robotically printed with 500 μg/mL influenza A capture antibody (MAB8800; EMD Millipore Corporation, Temecula, CA, USA, specific to IAV NP) in a volume of 1.2 nL per sensor using the sci-FLEXARRAYER S5 (Scienion, Germany; **Supplementary Figure S2**). For the control reactions bovine serum albumin (BSA; 1 mg/mL) and biotinylated bovine serum albumin (biotin-BSA; 1 mg/mL) were similarly printed onto GMR sensors. The 8 × 8 sensor array were divided into three regions (**Supplementary Figure S2**). Four columns (32 sensors) were spotted with influenza A capture antibody, two columns (16 sensors) with biotin-BSA, and the rest two columns (16 sensors) with BSA. Printed chips were incubated at 4°C for 12 h under a relative humidity of ∼97%. A bottomless reaction well made of polymethyl methacrylate (PMMA) was attached onto chip centered at the sensor area. This reaction well can hold as much as 100 μL liquid. Next, the sensor area was rinsed with PBST [0.05% tween 20 in phosphate buffered saline (PBS)] for three cycles to remove unbound biomolecules. To block any potential binding sites on the sensor, 100 μL of 10 mg/mL BSA was added to the reaction well and incubated at room temperature for 30 min. After removing BSA and washing the sensor area with 100 μL of PBST for three cycles, 100 μL of antigen (heat inactivated virus) of different concentrations were added to the reaction well and incubated at room temperature for 1 h. After washing the sensor area with 100 μL of PBST for three cycles, 100 μL of 5 μg/mL biotinylated detection antibody (MAB8257B, EMD Millipore Corporation, Temecula, CA, USA, a mouse anti influenza A monoclonal antibody specific for NP) was added and incubated at room temperature for another 1 h. Subsequently, detection antibody was aspirated and sensor area was rinsed with PBST for three cycles. Chips were kept at 4°C, 97% humidity condition before real-time testing. In order to detect all IAV subtypes, the capture and detection antibodies specific to influenza A NP were used. These antibodies were certified by the manufacturer as influenza A specific and the detection antibody was not shown to cross react with influenza B or other respiratory viruses.

### Detection Principle and Signal Flow

A sandwich assay structure (Srinivasan et al., 2009, 2011) used in this study is illustrated in **Figure 2B**. The detailed detection architecture is shown in **Figure 2C**. First, capture antibody was immobilized on the GMR sensor, then the antigen and biotinylated detection antibody were added successively and allowed to bind. Finally, streptavidin labeled MNPs (Miltenyi Biotec, Inc., Auburn, CA, USA; Catalog No. 130-048-101) were added and specifically bound to the near surface of sensor through the biotin-streptavidin interaction. Number of bound MNPs is proportional to number of target antigen. It is worthwhile to mention that, since there are 64 sensors in one GMR chip, it is possible to detect 64 types of biomolecules in one test.

In a bench top system, a probe station with 17 × 4 pin array (**Supplementary Figure S2**) is connected to the pads of GMR chip (Wang et al., 2015). An alternating current with frequency of 1000 Hz flows through the main bus. An in-plane magnetic field with amplitude of 30 Oe, frequency of 50 Hz is applied along the minor axis direction. A Digital Acquisition card (DAQ, NI USB-6289, 18-Bit, 625 kS/s) collects analog signals from side tones at 950 and 1050 Hz and carries out fast Fourier transform (FFT) before sending the data points back. It takes 1 s to collect one data points on one sensor, since there are 64 sensors in one GMR chip; it takes 1 min to go through all the sensors. Signals are extracted from the background noise using a Wheatstone bridge and then amplified by low-noise, low-distortion instrumentation amplifier (INA163, Texas Instruments).

### Enzyme Linked Immunosorbent Assay

Microtiter plates (Corning, Inc., Corning, NY, USA) were coated with 100 μL of 3 μg/mL anti-influenza A monoclonal antibody (MAB8800; EMD Millipore Corporation, Temecula, CA, USA) specific for influenza A NP. After overnight incubation at 4°C, the wells were blocked with 5% skim milk in PBS for 2 h at room temperature. 100 μL of heat inactivated virus diluted in sample diluent (3% BSA in PBS) was then added and incubated for 1 h at 37°C. After washing the wells three times with wash buffer (0.05% tween 20 in PBS), 100 μL of 1 μg/mL biotinylated anti-influenza A monoclonal antibody (MAB8257B; EMD Millipore Corporation, Temecula, CA, USA) was added and incubated for 1 h at room temperature. Wells were washed three times with wash buffer and incubated for 30 min at room temperature with 100 μL of 1:1000 diluted Pierce high sensitivity streptavidin- horseradish peroxidase (HRP; Thermo scientific, Rockford, IL, USA). After washing the wells three times with wash buffer, 100 μL of one step ultra TMB (Thermo scientific, Rockford, IL, USA) was added and the reaction was stopped after 30 min incubation at room temperature by adding 100 μL of 1 N H_2_SO_4_. The absorbance at 450 nm was measured by microtiter plate reader (Thermo Scientific). The cut off value was calculated as mean of negative control multiplied by 2.

## Results

**FIGURE 3:**
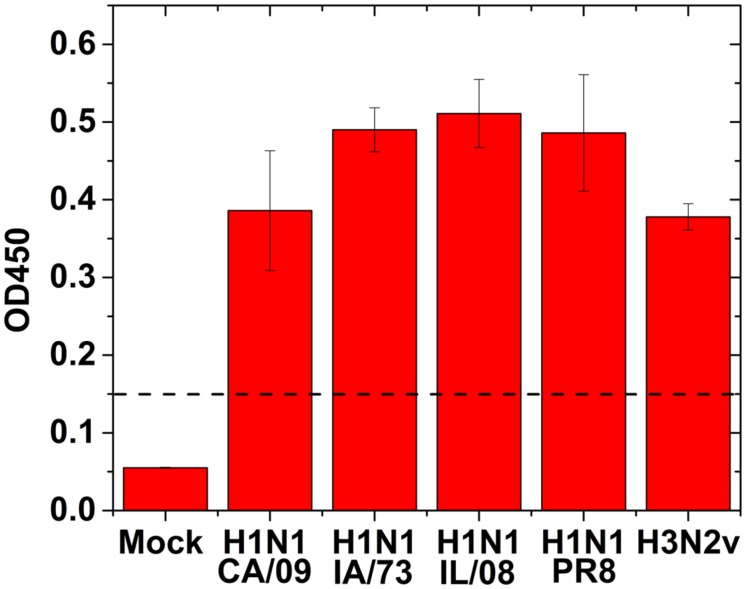
**Direct antigen capture ELISA for influenza virus detection using human and swine isolates of IAV.** 100 μl of 1:100 dilution of the virus (1.0 × 10^3^ TCID_50_/mL) were added to the wells for testing. Mock: no virus. Dotted line indicates the cut off value. Error bars represent SEM.

To validate the IAV specific antibodies to use in GMR biosensor assay, we performed antigen capture ELISA using human or swine isolates of IAV. All the IAV strains tested showed positive result by ELISA (**Figure 3**) suggesting that these antibodies are capable of detecting multiple IAV strains.

**FIGURE 4:**
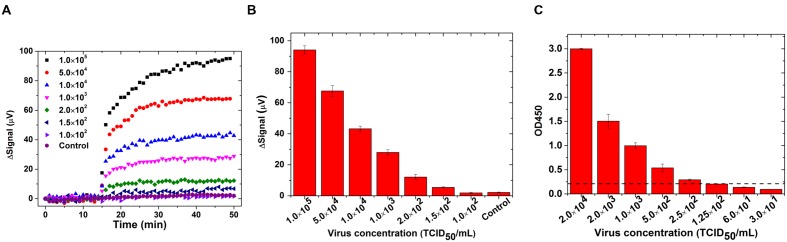
**Giant magnetoresistance biosensor showed higher sensitivity than ELISA for detection of IAV.** Swine IAV strain H3N2v or control (mock) were treated with 1% IGEPAL CA-630 to disrupt virus particle and used for detection by GMR biosensor and ELISA. **(A)** Binding curves in real-time on GMR biosensor; **(B)** Signals averaged over the last 10 data points from different concentrations of IAV and negative control (mock) in GMR biosensor and; **(C)** Antigen capture ELISA with different concentrations of IAV. Dotted line indicates the cut off value. Error bars represent SEM.

The real-time binding curves for influenza biosensor are shown in **Figure 4A**. Initially 30 μL of PBS was preloaded into the reaction well. After 15 min of stabilization, 30 μL MNP solution was added into reaction and signals were collected for another 35 min. Signals from reactions with different concentrations of virus increased immediately after addition of MNP solution, which indicates a real-time binding of MNPs onto GMR sensors. Furthermore, the signal increased with increasing concentration of virus. The averaged signal from 1.5 × 10^2^ TCID_50_/mL viruses is 5.45 μV, and it goes up with increased virus concentration and reaches to 94.1 μV for 1.0 × 10^5^ TCID_50_/mL virus (**Figure 4B**). The signal from negative control group did not show any obvious rise and the averaged signal was 2.2 μV, indicating that the signals are specific to IAV. The cut-off value for distinguishing positive from negative was set as 3.0 μV, since the mean value of negative control plus three times standard deviation of the mean is 3.0. As the signal from 1.0 × 10^2^ TCID_50_/mL viruses was below 3.0 μV (1.52 ± 0.37 μV), the detection limit of GMR sensor was estimated as 1.5 × 10^2^ TCID_50_/mL. We compared GMR biosensor assay for influenza virus with antigen capture ELISA and found that the GMR biosensor was more sensitive than ELISA. The limit of detection of antigen capture ELISA using these monoclonal antibodies was 2.5 × 10^2^ TCID_50_/mL (**Figure 4C**).

**FIGURE 5:**
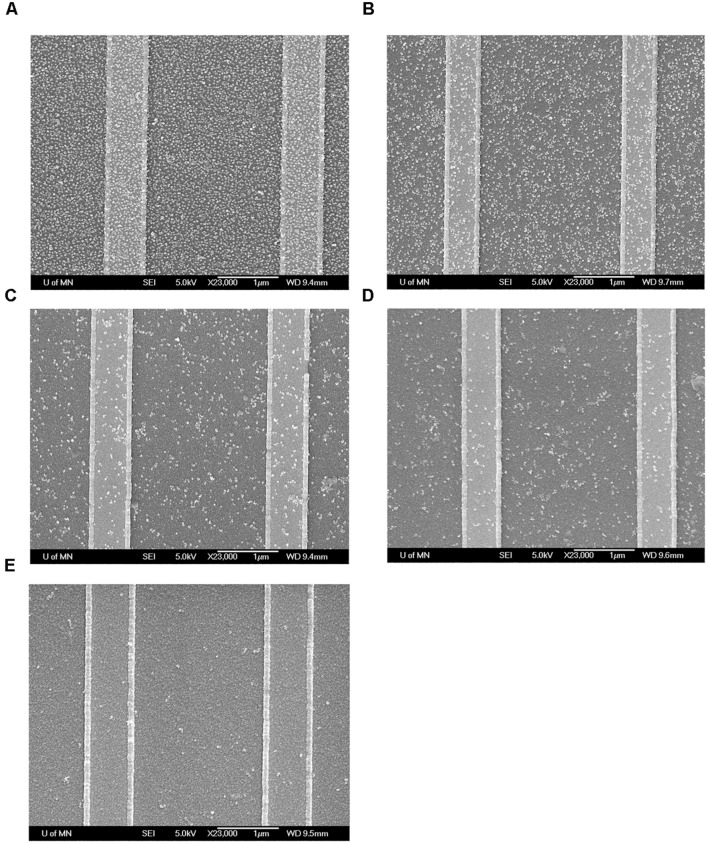
**Field-emission gun scanning electron microscopy (FEG-SEM) images of MNPs bound onto GMR sensors after test.** **(A)** Biotin-BSA positive control; **(B)** 1.0 × 10^4^ TCID_50_/mL virus; **(C)** 1.0 × 10^3^ TCID_50_/mL virus; **(D)** 2.0 × 10^2^ TCID_50_/mL virus; **(E)** BSA negative control.

To further confirm the binding of MNPs to the GMR sensor, these chips were investigated by field-emission gun scanning electron microscopy (FEG-SEM) at the Characterization Facility, University of Minnesota. The GMR chips were rinsed with DI water to wash away unbound MNPs and dried by nitrogen gas. The chips were then coated with 50 Å of Platinum (Pt) and observed by FEG-SEM. As shown in **Figure 5**, number of bound MNPs per unit area increases as the concentration of influenza virus increases.

## Discussion

This work extends the application of GMR-based assay for virus detection. In this study, a sensitive GMR biosensor was developed for the detection of influenza virus. We selected viral NP as target antigen to detect all strains of IAV, since large scale sequence analysis of this viral protein showed high degree of conservation among different subtypes of IAV from multiple hosts and lineages (Kukol and Hughes, 2014). As NP is localized within the virus particle, a non-ionic, non-denaturing detergent IGEPAL CA-630 was used to disrupt the virus particles in the sample. The results demonstrated that the GMR biosensor is able to detect viral concentrations ranging from 1.5 × 10^2^ to 1.0 × 10^5^ TCID_50_/mL. This is relevant to nasal samples of infected swine, which has been reported to contain 10^3^ to 10^5^ TCID_50_/mL viral particles (Lekcharoensuk et al., 2006). In addition, our results showed that the GMR biosensor is more sensitive than ELISA. Therefore, GMR biosensor assay has a potential for diagnosis and monitoring of influenza virus. In this study, we used purified virus diluted in buffer for the assay. Further work is required to address the effect of sample matrix on the sensitivity and specificity of GMR sensor. Although, detection antibody was not reported to cross react with influenza B or other respiratory viruses, further validation of GMR biosensor using these related viruses as well as multiple strains of swine influenza viruses are needed to assess the practical application of this technique.

Although, IAV H3N2v was used as a representative influenza virus in our GMR biosensor system, this assay can be further extended to other viruses and infectious agents. Since there are 64 sensor arrays in one GMR chip, this assay can be further optimized to detect different subtypes of influenza viruses in a single test or simultaneously detect different pathogens from single sample. The assay is simple with minimum sample preparation, although GMR biosensor surface functionalization and antibody immobilization on the sensor requires time and labor. Protein incubation, sample handling and washing could be improved by integrating with the well-developed microfluidic channel platform. In addition, it is possible to integrate this assay into portable, hand held device for on-site application.

## Author Contributions

J-PW and AP conceptualized this research. VK and KW planned the experiments. KW carried out the GMR biosensing experiments. VK carried out the ELISA reference experiments. The manuscript was written through contributions of all authors. All authors have given approval to the final version.

## Conflict of Interest Statement

Dr. J-PW has equity and royalty interests in, and serves on the Board of Directors and the Scientific Advisory Board, for Zepto Life Technology LLC, a company involved in the commercialization of GMR Biosensing technology. The University of Minnesota also has equity and royalty interests in Zepto Life Tech LLC. These interests have been reviewed and managed by the University of Minnesota in accordance with its Conflict of Interest policies.
