# Supplementary material for: Giant Magnetoresistance-based Biosensor for Detection of Influenza A Virus
Source: Front Microbiol. 2016 Mar 29;7:400. doi: 10.3389/fmicb.2016.00400 (PMC4809872; doi:10.3389/fmicb.2016.00400)
Supplement: Supplementary file 1 [file Presentation_1.PDF]

Supplementary Material

Giant Magnetoresistance based Biosensor for Detection of Influenza A Virus

Venkatramana D. Krishna[1‡], Kai Wu[2‡], Andres Perez[1]*, and Jian-Ping Wang[2]*

[1]Department of Veterinary Population Medicine, College of Veterinary Medicine, University of Minnesota, St. Paul, MN, USA.

[2]Department of Electrical and Computer Engineering, University of Minnesota, Minneapolis, MN, USA.

*Corresponding authors: Jian-Ping Wang, jpwang@umn.edu; Andres Perez, aperez@umn.edu

[‡]These authors contributed equally to this work

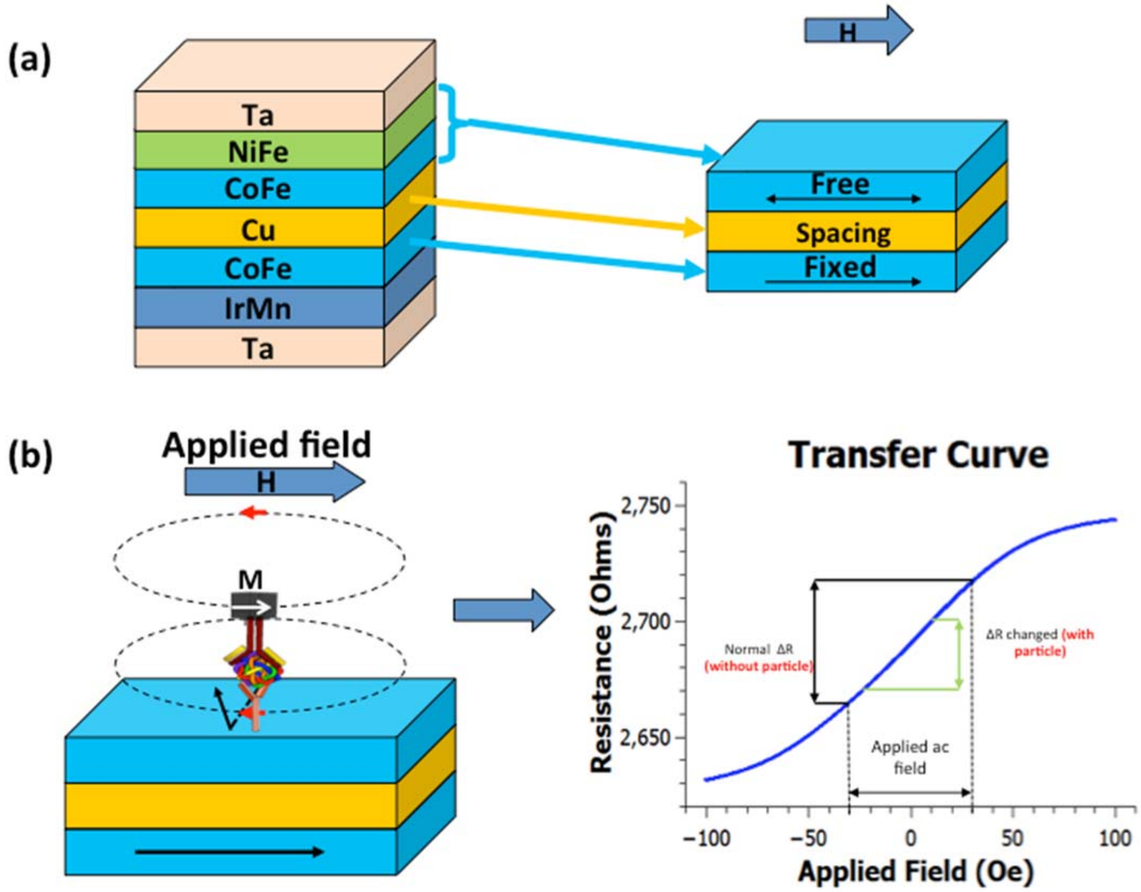

Supplementary Figure 1. (a) Free layer, spacing, and fixed layers in a GMR biosensor; (b) Mechanism of GMR based biosensing. Giant magnetoresistance is a phenomenon that the resistance of multi-layer thin film structures (alternating ferromagnetic and non-magnetic layers) changes in response to an applied magnetic field.

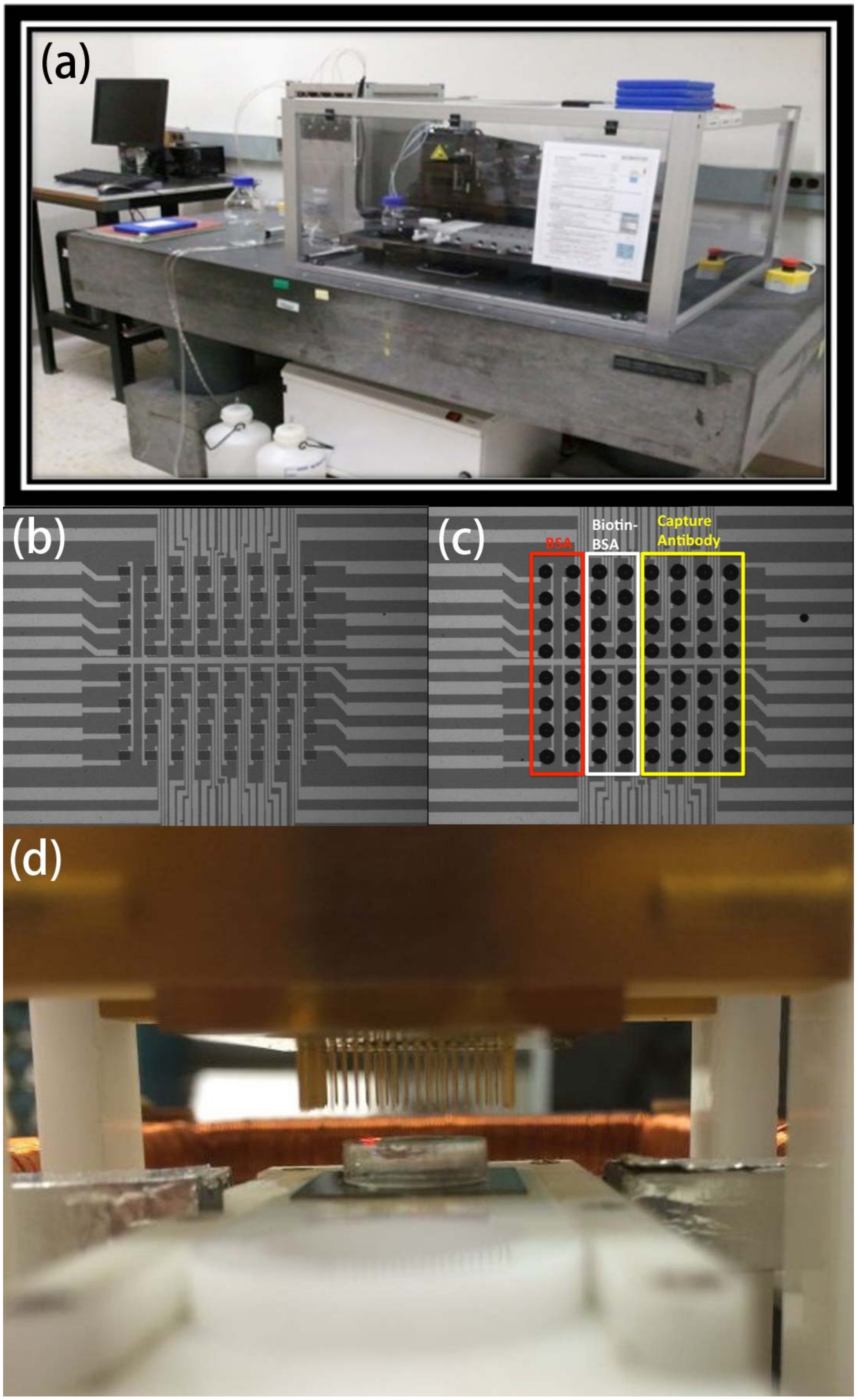

Supplementary Figure 2. (a) sci-FLEXARRAYER S5; (b) 8 × 8 sensor array before printing; (c) 8 × 8 sensor array after printing, four columns are spotted with influenza A capture antibody,

two columns with biotin-BSA, and the rest two columns with BSA; (d) probe station with 17 × 4 pin array connected to pads on GMR chip.
